# Supplementary material for: Thermally Crosslinked Hydrogen-Bonded Organic Framework Membranes for Highly Selective Ion Separation
Source: Molecules. 2023 Feb 26;28(5):2173. doi: 10.3390/molecules28052173 (PMC10004400; doi:10.3390/molecules28052173)

checkCIF/PLATON report

You have not supplied any structure factors. As a result the full set of tests cannot be run.

THIS REPORT IS FOR GUIDANCE ONLY. IF USED AS PART OF A REVIEW PROCEDURE FOR PUBLICATION, IT SHOULD NOT REPLACE THE EXPERTISE OF AN EXPERIENCED CRYSTALLOGRAPHIC REFEREE.

No syntax errors found. CIF dictionary Interpreting this report

Datablock: ga_210122a_a

```
Bond precision:   C-C = 0.0023 A          Wavelength=1.34138

Cell:              a=3.6813(1)      b=11.5900(4)      c=15.8935(5)
                   alpha=90         beta=90           gamma=90
Temperature:       173 K

                Calculated                    Reported
Volume          678.12(4)                     678.12(4)
Space group     P n n m                       P n n m
Hall group      -P 2 2n                       -P 2 2n
Moiety formula  C12 H12 N10, 2(H2 O)          C12 H12 N10, H4 O2
Sum formula     C12 H16 N10 O2                C12 H16 N10 O2
Mr              332.35                        332.35
Dx,g cm-3       1.628                         1.628
Z               2                             2
Mu (mm-1)       0.643                         0.648
F000            348.0                         348.0
F000'           348.84
h,k,lmax        4,14,20                       4,14,20
Nref            761                           760
Tmin,Tmax       0.954,0.968                   0.647,0.751
Tmin'           0.931

Correction method= # Reported T Limits: Tmin=0.647 Tmax=0.751
AbsCorr = NONE

Data completeness= 0.999           Theta(max)= 58.107

R(reflections)= 0.0568( 657)                         wR2(reflections)=
                                                     0.1641( 760)
S = 1.195                  Npar= 61
```

The following ALERTS were generated. Each ALERT has the format
test-name_ALERT_alert-type_alert-level.
Click on the hyperlinks for more details of the test.

Alert level B

```
DIFMN02_ALERT_2_B  The minimum difference density is < -0.1*ZMAX*1.00
            _refine_diff_density_min given =     -0.899
            Test value =     -0.800
PLAT098_ALERT_2_B Large Reported Min.  (Negative) Residual Density      -0.90 eA-3
```

Alert level C

```
DIFMN03_ALERT_1_C  The minimum difference density is < -0.1*ZMAX*0.75
            The relevant atom site should be identified.
DIFMX02_ALERT_1_C  The maximum difference density is > 0.1*ZMAX*0.75
            The relevant atom site should be identified.
PLAT042_ALERT_1_C Calc. and Reported MoietyFormula Strings  Differ     Please Check
PLAT097_ALERT_2_C Large Reported Max.  (Positive) Residual Density       0.80 eA-3
```

Alert level G

```
ABSMU01_ALERT_1_G  Calculation of _exptl_absorpt_correction_mu
                not performed for this radiation type.
PLAT007_ALERT_5_G Number of Unrefined Donor-H Atoms .............          4 Report
PLAT012_ALERT_1_G No       _shelx_res_checksum Found in CIF ......     Please Check
PLAT300_ALERT_4_G Atom Site Occupancy of H1C        Constrained at        0.5 Check
PLAT300_ALERT_4_G Atom Site Occupancy of H1D        Constrained at        0.5 Check
PLAT417_ALERT_2_G Short Inter D-H..H-D       H1A      ..H1C      .       1.71 Ang.
                                             1+x,y,1-z  =      6_656 Check
PLAT883_ALERT_1_G No Info/Value for _atom_sites_solution_primary .     Please Do !
PLAT984_ALERT_1_G The  C-f'=    0.0150 Deviates from the B&C-Value     0.0137 Check
PLAT984_ALERT_1_G The  O-f'=    0.0410 Deviates from the B&C-Value     0.0389 Check
```

0 **ALERT level A** = Most likely a serious problem - resolve or explain
2 **ALERT level B** = A potentially serious problem, consider carefully
4 **ALERT level C** = Check. Ensure it is not caused by an omission or oversight
9 **ALERT level G** = General information/check it is not something unexpected

8 ALERT type 1 CIF construction/syntax error, inconsistent or missing data
4 ALERT type 2 Indicator that the structure model may be wrong or deficient
0 ALERT type 3 Indicator that the structure quality may be low
2 ALERT type 4 Improvement, methodology, query or suggestion
1 ALERT type 5 Informative message, check

It is advisable to attempt to resolve as many as possible of the alerts in all categories. Often the minor alerts point to easily fixed oversights, errors and omissions in your CIF or refinement strategy, so attention to these fine details can be worthwhile. In order to resolve some of the more serious problems it may be necessary to carry out additional measurements or structure refinements. However, the purpose of your study may justify the reported deviations and the more serious of these should normally be commented upon in the discussion or experimental section of a paper or in the "special_details" fields of the CIF. checkCIF was carefully designed to identify outliers and unusual parameters, but every test has its limitations and alerts that are not important in a particular case may appear. Conversely, the absence of alerts does not guarantee there are no aspects of the results needing attention. It is up to the individual to critically assess their own results and, if necessary, seek expert advice.

Publication of your CIF in IUCr journals

A basic structural check has been run on your CIF. These basic checks will be run on all CIFs submitted for publication in IUCr journals (*Acta Crystallographica*, *Journal of Applied Crystallography*, *Journal of Synchrotron Radiation*); however, if you intend to submit to *Acta Crystallographica Section C* or *E* or *IUCrData*, you should make sure that full publication checks are run on the final version of your CIF prior to submission.

Publication of your CIF in other journals

Please refer to the *Notes for Authors* of the relevant journal for any special instructions relating to CIF submission.

PLATON version of 28/11/2022; check.def file version of 28/11/2022

Datablock ga_210122a_a - ellipsoid plot

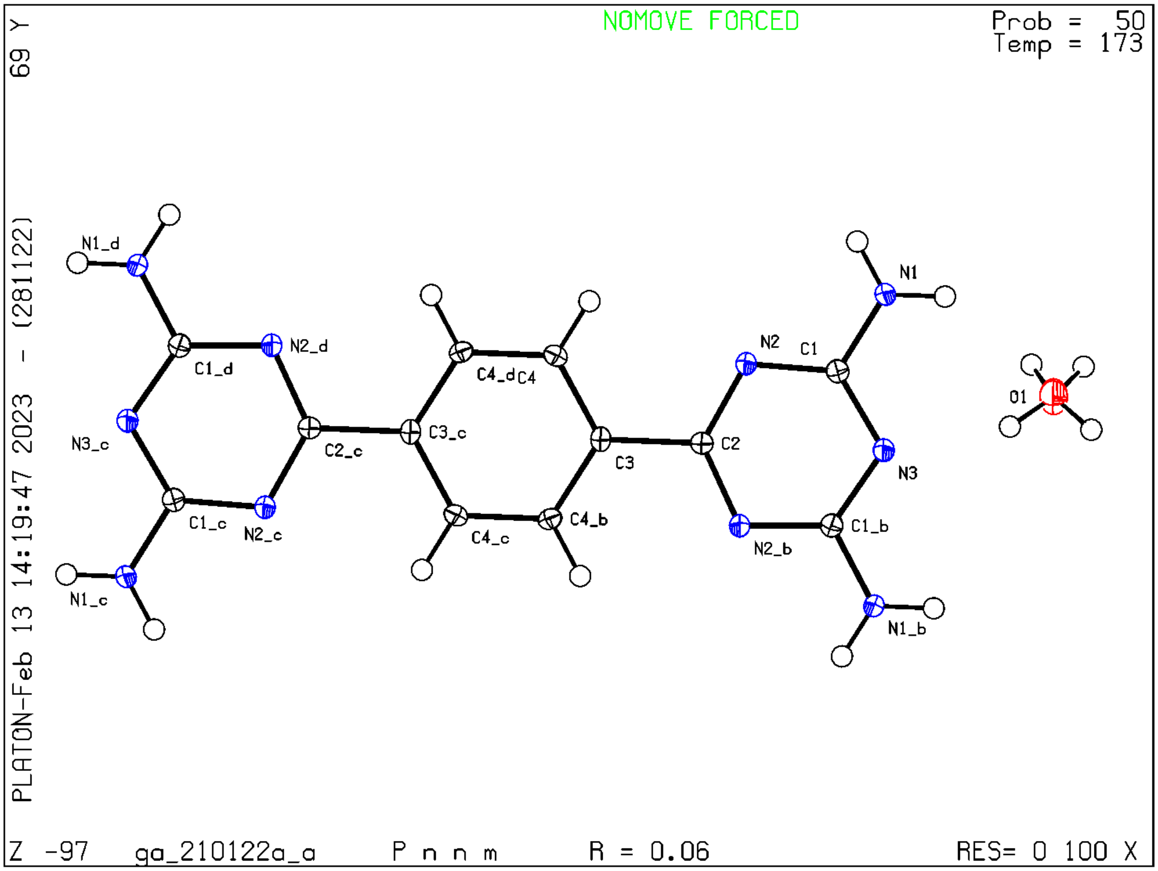

Supplement: Supplementary file 1 [file molecules-28-02173-s001.zip › File S1 FDU-HOF-1 Checkcif.pdf]
