# Supplementary material for: Thermally Crosslinked Hydrogen-Bonded Organic Framework Membranes for Highly Selective Ion Separation
Source: Molecules. 2023 Feb 26;28(5):2173. doi: 10.3390/molecules28052173 (PMC10004400; doi:10.3390/molecules28052173)
Supplement: Supplementary file 1 [file molecules-28-02173-s001.zip › Supporting Information.pdf]

Supporting Information

Thermally-Crosslinked Hydrogen-Bonded Organic Framework Membranes for Highly Selective Ion Separation

Xiyu Song,[1,2] Chen Wang,[2] Xiangyu Gao,[2] Yao Wang,[2] Rui Xu,[2] Jian Wang,[1,*] Peng Li[2,*]

[1] School of Physical Education and Coaching, Shanghai University of Sport, Shanghai, 200438, China

[2] Department of Chemistry and Shanghai Key Laboratory of Molecular Catalysis and Innovative Materials, Fudan University, Shanghai 200437, China

* Correspondence: ilsrwang@163.com (J.W.); penglichem@fudan.edu.cn (P.L.)

Table S1. Crystal data and structure refinement for FDU-HOF-1

Name	FDU-HOF-1
Empirical formula	$C_{12}H_{16}N_{10}O_2$
CCDC number	2238476
Formula weight	332.35
Temperature/K	173
Crystal system	orthorhombic
Space group	Pnnm
a/ Å	3.6813 (1)
b/ Å	11.5900 (4)
c/ Å	15.8935 (5)
α°	90
β°	90
γ°	90
Volume/Å^3	678.12 (4)
Z	2
D_c (g·cm^{-3})	1.628
μ (mm^{-1})	0.643
F(000)	348.0
Crystal size (mm)	0.11 × 0.06 × 0.05
θ range for data collection (°)	4.107-58.107
Refinement method	Least Squares

Data/restraints/parameters	760/0/63
Goodness-of-fit on F^2	1.247

Table S2. Different Connolly radius of FDU-HOF-1 of occupied volume, free volume and surface area.

Connolly radius (Å)	Occupied Volume (Å^3)	Free Volume (Å^3)	Surface □rea (Å^2)
0.6	564.07	114.05	166.78
0.9	593.35	84.76	123.75
1.3	616.85	61.26	106.04

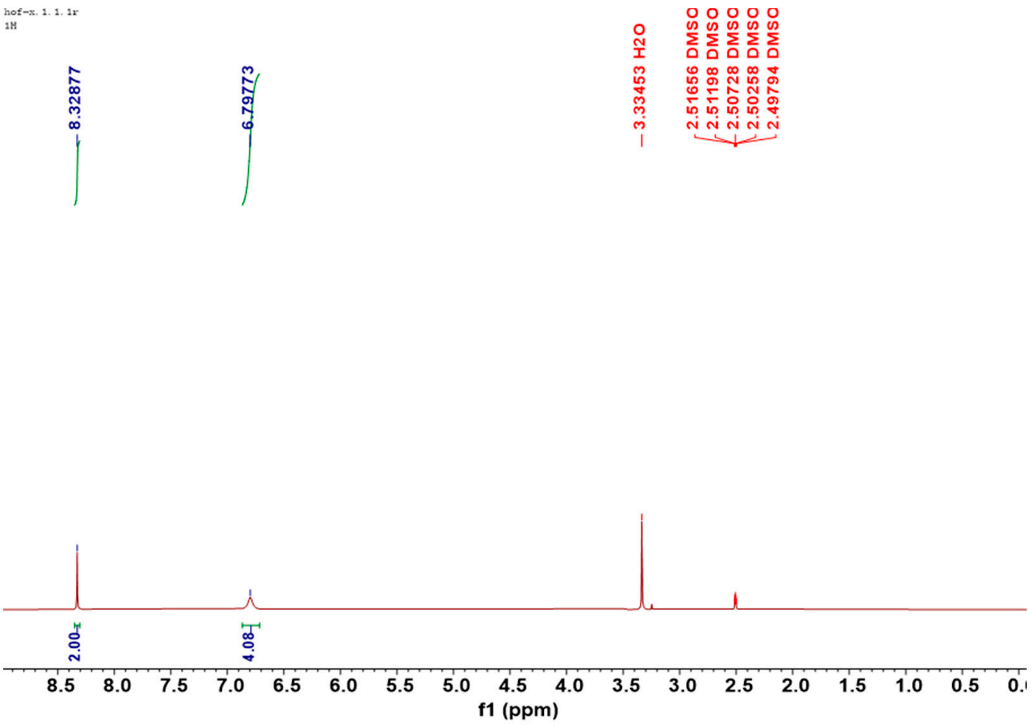

Figure S1. ^{1}H NMR spectrum of BD□T.

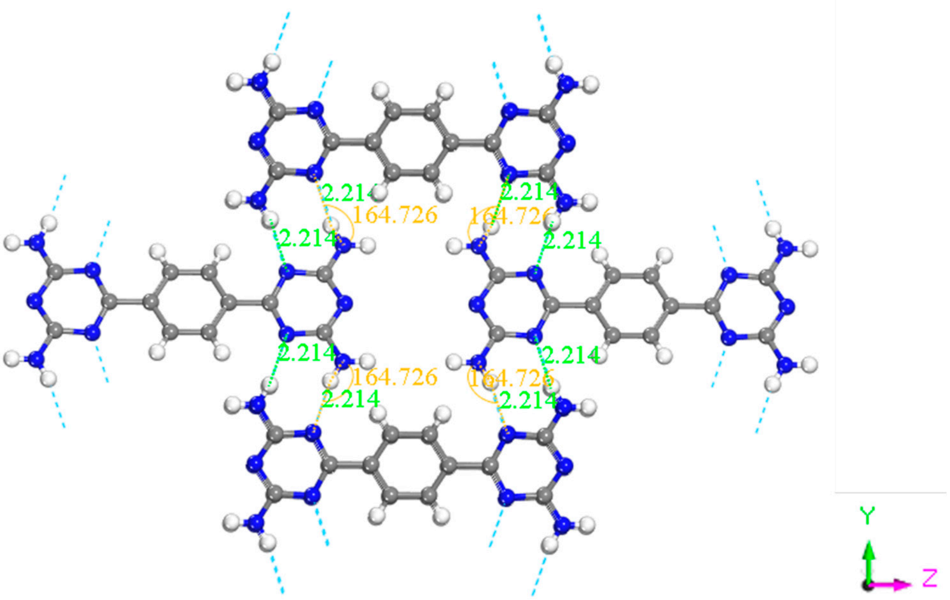

Figure S2. H-bonded distance and angle of BD□T ligand of FDU-HOF-1.

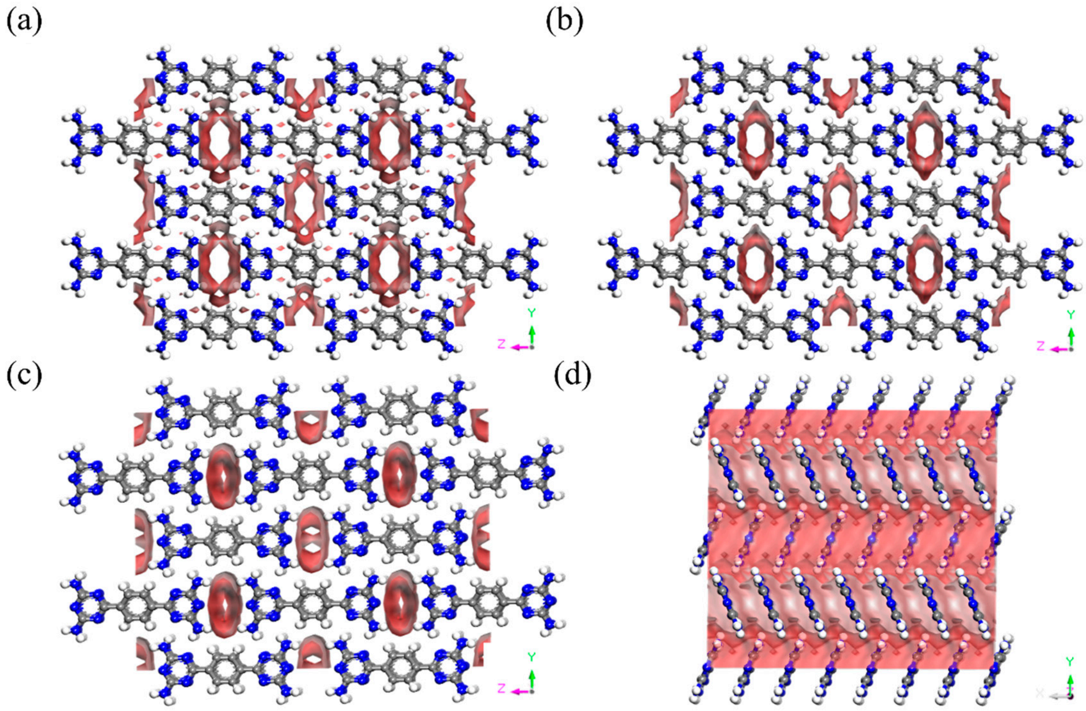

Figure S3. Pore structure at (a) 0.6 Å, (b) 0.9 Å and (c) 1.3 Å probe molecular sizes of FDU-HOF-1 in Materials Studio 2019. (d) Pore structure display along z-axis of FDU-HOF-1.

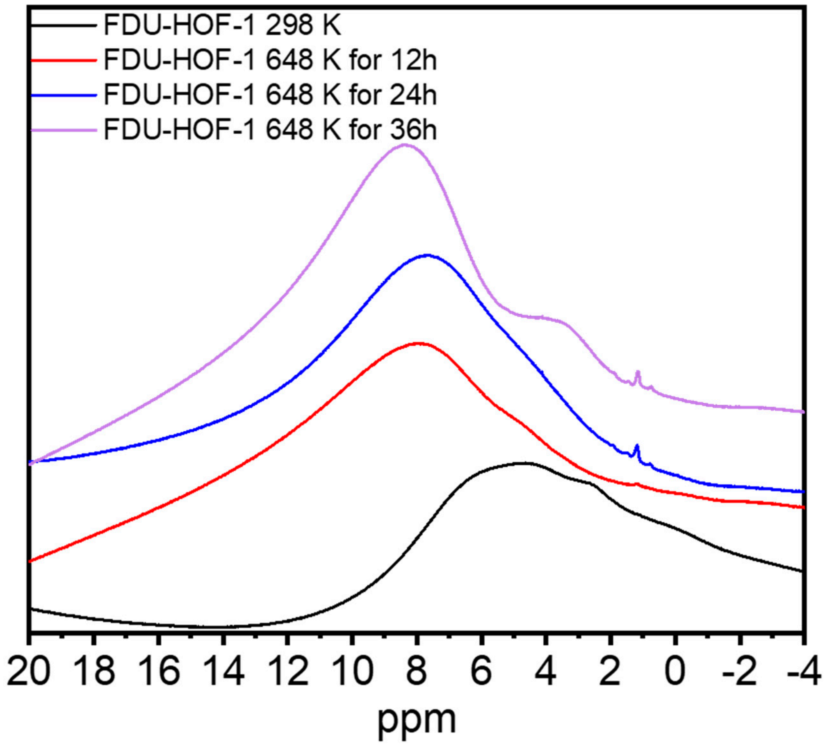

Figure S4. Solid-state ^{1}H NMR spectrums of FDU-HOF-1.

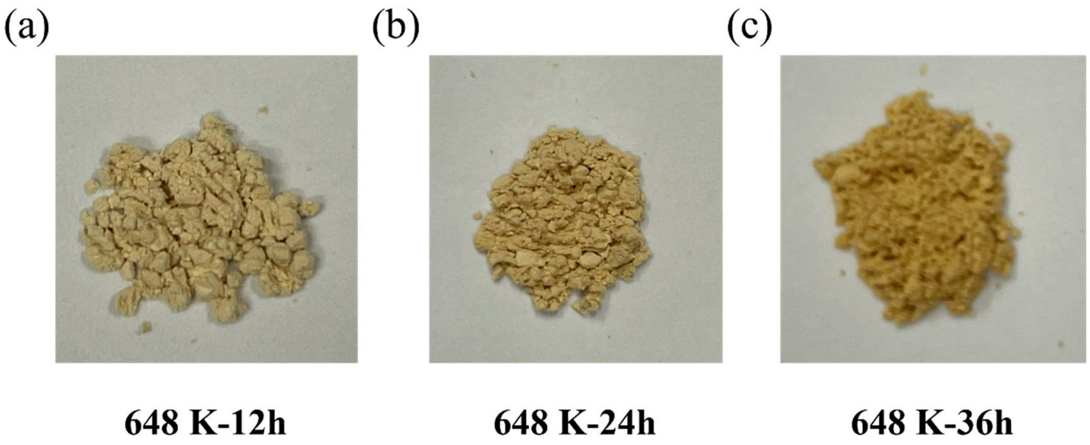

Figure S5. Photographs of FDU-HOF-1 powder after being treated under 648 K of (a) 12 h, (b) 24 h and (c) 36 h.
